# Deformational plagiocephaly: The case for an evolutionary mismatch

**DOI:** 10.1093/emph/eoy019

**Published:** 2018-08-08

**Authors:** Herbert Renz-Polster, Freia De Bock

**Affiliations:** 1 Mannheim Institute of Public Health, Social and Preventive Medicine, University Medicine Mannheim, Heidelberg University, Ludolf-Krehl-Straße Mannheim, Germany; 2 Center for Child Neurology, Theobald-Christ-Straße 16, Frankfurt a.M., Germany

**Keywords:** flat head syndrome, positional plagiocephaly, evolutionary mismatch, supine sleep position, cranial flattening, Kindchenschema

## Abstract

Lay Summary: In industrialized societies some babies develop flattening of the back part of their head. It is thought that this comes from sleeping supine, which has been shown to be the safest option for babies. However, this explanation cannot be correct from an evolutionary standpoint: why should safe sleep come at the cost of a misshaped head?

Babies in industrialized societies are generally healthy. The medical problems they may be afflicted with are usually well understood. Deformational plagiocephaly presents a notable exception. In many industrialized countries, one in six babies shows posterior flattening of the skull—a feature noteworthy from an evolutionary perspective as the well rounded cranium is part of the ‘Kindchenschema’ evolved to secure care for the infant. It is commonly held that the deformation of the posterior cranium occurs as a consequence of the supine sleep position, now advocated as the safest sleep position for babies by medical experts. This explanation, however, does not fare well in the light of evolutionary theory: why should safe sleep come at the cost of a social handicap? Here, we present an alternative hypothesis that is grounded on evolutionary mismatch theory and exemplifies how evolutionary reasoning can help clarify medical conditions relevant to today’s public health.

## INTRODUCTION

About 15–20% of the 4- to 6-month-old infants in industrialized societies are now affected by symmetrical or asymmetrical flattening of their posterior cranium [1]. Among a large variety of intrinsic and extrinsic determinants (like male gender, limited neck rotation or preference in head position, first-born child, lower level of activity, and lack of tummy time), the supine sleeping position, advocated since the early 1990s as part of the ‘back to sleep’ campaign for the prevention of sudden infant death syndrome (SIDS), figures prominently as a possible explanation for the development of deformational plagiocephaly (DP) [2–4].

At first glance, the temporal association as well as biophysical considerations make the supine sleeping position an appealing explanation. Indeed, the ‘epidemic’ of DP started shortly after the prone sleeping position had been dismissed as unsafe by the pediatric community in the 1990s [5–7]. Also, mechanical factors automatically come into focus as supine positioning clearly goes along with gravitational pressure to the back of the skull—a relatively soft bony structure in the first few weeks and months of life, which may easily be deformed when overly strained.

However, there are two reasons that make this simple hypothesis an unlikely explanation—one reason is grounded on empirical evidence, the other is grounded on evolutionary theory.

## SHORTCOMINGS OF THE CURRENT EXPLANATION

The empirical caveat comes from the aggregated research record on the role of the supine sleeping position in the development of DP. In our recent review of empirical studies on the etiology of DP published in the major biomedical databases between 1985 and 2016, supine sleep position emerged as a significant determinant of DP in only 6 out of the 14 relevant studies—the other 8 studies did not attribute significance to this exposure [4].

The theoretical caveat comes from evolutionary considerations. In order to secure resources human infants rely on a set of bodily and especially facial features that signal ‘cuteness’ or attractiveness: a symmetric, round face, high forehead, big eyes, small nose, retreating chin and a rounded posterior part of the head [8]. This pattern of appealing childlike attributes has been called ‘Kindchenschema’ by the German ethologist Konrad Lorenz [9]. It is thought to motivate potential caregivers to protect and care for the baby as it enters a developmental stage in which increasing amounts of support (including supplementary feeding) can be mustered by caregivers other than the (breastfeeding) mother [10, 11]. Indeed, babies around the world get more appealing as they approach the middle of their first year of life. Data on infant handling, adoption preferences and developmental outcomes show that the Kindchenschema is not a trivial concept but does have implications for the amount and quality of infant care through adults even in today’s societies: cuteness is an important developmental resource [12–15].

Another highly important prerequisite for survival, of course, is safe sleep. As both epidemiological data and experimental evidence from sleep lab research show, the supine sleeping position has to be considered the safest option compared with side and prone positioning and is therefore officially recommended by medical authorities worldwide [16]. The safety argument is considered paramount especially for the first 6 months of life where the incidence of SIDS is the highest [17].

It is exactly this developmental period, during which some of the babies, positioned supine to avoid potentially lethal risks, develop flattening of their posterior cranium—a condition also associated with developmental risks as it interferes with their signaling of health and vigor. After all, DP constitutes a multi-pronged attack on the ‘Kindchenschema’ as it not only flattens the posterior cranium but also induces compensatory shifts in the bony facial architecture, e.g. by flattening the forehead, by introducing facial asymmetry or by making the eyes appear protruded [18].

So clearly, the relationship between safe sleep and the development of DP is contradictory: if the supine sleeping position is the safest position for infant sleep—why should it result in a social handicap for a significant proportion of babies? After all, safe sleep is a highly favored outcome from an evolutionary perspective—but so is normal skull and facial architecture.

## THE NEED FOR A NEW HYPOTHESIS

Both the contradictory empirical record on the determinants of DP as well as the contradictory relationship between safe sleep and skull deformation support the conclusion that positioning babies on their backs cannot be a complete and sufficient explanation for DP.

We therefore propose an alternative hypothesis which may be able to resolve the apparent contradictions. Rather than seeing DP as a medical or developmental disorder in its own right we propose to consider DP a manifestation of a biological–environmental mismatch. In our opinion DP needs to be viewed as a ‘cost’ of care practices in current industrialized environments that are different from those for which human infants have been prepared for through evolutionary history.

As a first step to defend our hypothesis we will take a closer look at the typical components of the current infant care environment potentially relevant to cranial shape development, and ask in which respect they could represent candidates for mismatch situations that interfere with normal head shape development. To make this theoretical investigation as complete as possible we are drawing on findings from multiple disciplines including sleep research and human ethology and cover the whole set of caregiver–infant interactions potentially relevant to head shape development, i.e. feeding, transport, and sleep behavior.

### Infant feeding and head shape development

All young babies invariably have been breastfed in the evolutionary context—there was no other way compatible with infant survival in the hunter-gatherer ecology. The typical head position while being nursed in all primates clearly is one of repetitive positional change—in part caused by the fact that the two breasts are only accessible through different body positions. Feeding time, from an evolutionary standpoint, has certainly not been associated with prolonged gravitational pressure to a specific portion of the skull. In contrast, bottlefeeding may well result in asymmetric or prolonged strain to the back of the skull, especially if a bottle is propped in a fixed position to facilitate sleep.

### Transport and head shape development

Babies in the evolutionary context have been carried by their caregivers—on their hips, in their arms, in slings, in devices manufactured from natural materials including fur, leather and plant material. This experience presumably not only contributes to muscular development of the neck, legs, and trunk but also places the infant in a secure yet exposed position that facilitates social learning from members of the caregiver’s social network. It therefore comes as no surprise that being carried appears as a biological adaptation in humans [19]. Indeed, the maturation of the infant’s hip joints is dependent on repetitive abduction of the femurs—a position typical for being carried. Also, when lifted up, infants universally assume the typical squatting position in preparation for being placed at a caregiver’s body. The gravitational impact of being carried has not been studied in detail but it has to be assumed that pressure on the same area of the skull is minimized as the physical impact varies depending on the child’s activity, its developmental abilities, as well as the caregiver’s activities while carrying.

In contrast, baby transport typical for industrialized countries (i.e. in car seats, prams, and strollers) is more likely to leave the baby’s head continuously exposed to gravity in the same position, sometimes even with the baby’s head against a hard surface, as in the case of some car seat models. Additionally, in some families, transport or carrying devices are being used for ‘parking’ the baby while adults engage in their activities—clearly translating into prolonged and uniform gravitational impact to the skull.

### Infant sleep and head shape development

The fact that sleep constitutes a human baby’s predominant daily behavior makes sleep an attractive field of investigation when it comes to assessing influences on the developing cranium of the human offspring. As mentioned earlier, the currently favored hypothesis of the development of DP indeed focuses on the position in which babies are put to sleep by their caregivers.

However, evidence from the sleep lab adds a more comprehensive perspective and points to the fact that the position in which a baby is put to sleep is only one of several influences that determine in which position she sleeps most of the time. Indeed, according to experimental research, infant sleep and infant sleep position differs significantly depending on the social context in which it happens. Starting in the 1990s infant sleep researchers have described noticeable characteristics for babies sleeping in proximity to their breastfeeding mothers—this ‘breastsleeping’ pattern is considered the evolved, species typical arrangement in humans by evolutionary biologists as well as anthropologists [20–23]. After all, babies placed alone for sleep would have been prone to be abducted by predators, to be bitten or stung by reptiles and insects, or would have suffered compromised thermoregulation in most climate zones.

Experimental sleep research is able to describe the evolved same surface cosleeping disposition in human infants in physiological details. When placed in proximity in the sleep laboratory, sleep stages tend to synchronize between the mother and her infant [10]. Also, on the part of the baby, sleep architecture changes—breastfed, bedsharing infants spend more time in ‘active’ sleep stages than babies sleeping in their own crib during their first months of life [24–26]. Concomitant with the higher proportion of active sleep—marked by higher muscle tone and more frequent arousals—motor activity during sleep increases in the bedsharing setting. Also, during dyadic sleep, babies not only have more wake periods and more frequent feeding sessions but also experience more passive re-positioning: as infrared videotaping shows, the nursing mother regularly checks on, touches, soothes and re-places her baby even while she is asleep—the preferred placement position for the infant being on its side or its back, i.e. positions that facilitate easy access to the breast (indeed, breastfed, bedsharing infants have been shown to spend more than half of their sleep time in the side sleeping position) [27, 28].

It could also be hypothesized that the whole package of evolutionarily typical parenting practices (breastfeeding, bedsharing, extensive parent-baby contact, baby transport on or at the caregiver’s body) may represent a maturational advantage in terms of musculoskeletal development—which in turn, by virtue of a more developed neck, arm and shoulder musculature, may temper the effect of being put down to sleep supine and/or may allow the baby to engage sooner or more successfully in ‘tummy time play’, which may also work against the development of DP.

All these evolutionarily typical influences—lighter sleep with more motor activity, more arousals, more repositioning, a mix of side and supine sleeping—translate into less prolonged and less uniform pressure to the back of the skull in the proximate sleep model. Therefore, to what extent sleep translates into ‘gravity time’, may be less a question of how the infant is placed by the caregiver for sleep onset, but rather a matter of social sleep ecology, i.e. where and with whom the baby sleeps.

From this vantage point, the biophysical model of head shape development central to the traditional hypothetical model of DP development needs to be enriched by an ecological component: sleeping on the back may be a risk factor for the development of DP—but only under certain circumstances that are capable of overwhelming the infant’s biological developmental capacities. In the context of this ecologically augmented model the supine sleeping position may or may not be a risk factor for the development of DP—depending on the environmental context in which sleep happens.

## THE CASE FOR A BIO-CULTURAL MISMATCH HYPOTHESIS

The above model and considerations lend theoretical support to our core assumption: instead of considering DP a developmental disorder, it may better be viewed as an artifact of infant care practices in industrialized countries, which all conspire to exert more uniform and prolonged pressure on the backs of their skulls:

- bottlefeeding instead of breastfeeding;
- transporting infants in fixed physical structures (prams, strollers, or car seats) instead of carrying them close to the human body;
- having infants sleep solitarily instead of bedsharing.

Testing this hypothesis against the current evidence in DP research yields the following picture:

### Breast- versus bottlefeeding

The hypothesis that feeding mode may play a role in the development of DP has been discussed in several reviews and commentaries over the last 20 years, in part based on empirical epidemiological work by Hutchison, Losee, and van Vlimmeren that shows an association between DP and bottlefeeding, fixed feeding positions while being bottle fed and bottle propping [29–31].

### Baby transport

Mode of transport as a risk factor for developing DP has been studied only in respect to the use of car seats, especially when used as sleep devices for daytime naps. Chadduck and Glasgow both found significant interactions between car seat use for sleep purposes and DP [32, 33]. The influence of stroller or pram use on the development of DP has not been reported in empirical studies as of yet, nor has the possibly preventive effect of babywearing been investigated.

### Sleep

Sleep has been considered relevant for the development of DP in several respects. As mentioned before, infant placement for sleep (supine versus prone) has been the most extensively mentioned and discussed exposure in the literature—and the reason why the rise in DP has been correlated with the ‘back to sleep’ campaign in countless reviews, editorials and other publications. However, the empirical evidence is contradictory and systematic reviews do not support a role of the supine sleep position as an independent risk factor for DP [1, 4]. Other factors impacting infant sleep have been subject to empirical investigation, including the influence of the sleep surface (e.g. firm mattresses—no significant interaction) and the use of swings, car seats or bouncy seats (no significant interaction except for very heavy use) [29, 34–36]. Alternate positioning or re-positioning as well as change in orientation of the bed has been studied as part of a multi-component prevention study, conclusions as to effectiveness are limited due to the complex nature of the intervention [37]. Solitary sleep as a potential contributing factor for the development of DP, however, has not been reported in the literature as of yet nor has it been studied in comparative designs. The same holds true for the influence of side-car cribs (also known as bed-side cots or ‘co-sleepers’), now ever more in use in young families.

## TESTING THE HYPOTHESIS EMPIRICALLY

It is not possible to draw enough data from the current literature when it comes to testing our assumption that DP may be an unintended effect of infant care practices in industrialized countries. Experimental studies—although feasible at least when it comes to comparing different modalities in infant transport—are lacking entirely.

How could the spotty picture be completed? For one, of course there is a need for more high quality epidemiological studies on the determinants of DP. Cross-cultural comparisons could also be of interest. According to our hypothesis, an inverse correlation between DP and traditional life styles should be expected: the more traditional the child rearing practices (same surface cosleeping, use of slings for baby transport) the less DP should be observed in infants—even if they routinely sleep on their backs or on their sides.

Another easy and potentially rewarding route of investigation could be a comparison within industrialized societies of different parenting styles. It is the impression of the authors that there is virtually no DP in infants in the bedsharing and baby-carrying ‘attachment parenting’ scene in Germany, but this personal observation, of course, needs to be substantiated in a well-planned study.

## CONCLUSION

We think that the considerations laid out in this publication are relevant for clinical practice as pediatricians thus far are in a less than optimal position: treatment options for the more severe cases of DP are cumbersome, expensive and not well supported by evidence [38]. Also, current surveys indicate that DP remains a public health challenge despite so many suggestions for its prevention [39]. The most problematic issue, however, is a practical one: there are only two studies that have examined primary prevention of DP in a rigorous fashion [37, 40]. None of these studies have included the most easily modifiable lifestyle variable, i.e. baby transport. There is not one single official recommendation that currently suggests infant wearing as a preventive measure against the development of DP [4]. So as of yet, pediatricians do not have sound answers to important questions of a great number of parents: how may DP in my baby be prevented? And what if my baby already shows a touch of DP: what can I do?

From theoretical considerations, the answer may well be look at how the baby sleeps, look at how it is being transported, and look at how it is being fed. Look for bio-cultural mismatch situations that may put the baby at a developmental disadvantage.
